# LONG-TERM PSYCHOLOGICAL CONSEQUENCES OF PARENTAL BEREAVEMENT PRIOR TO MID-LIFE: VOLUNTEERING HELPS

**DOI:** 10.1093/geroni/igac059.1368

**Published:** 2022-12-20

**Authors:** Meng Huo, Kyungmin Kim

**Affiliations:** University of California, Davis, DAVIS, California, United States; Seoul National University, Seoul, Seoul-t'ukpyolsi, Republic of Korea

## Abstract

Losing a child prior to midlife may be a uniquely traumatic event that continues to compromise parents’ well-being in later life. This study compared psychological well-being between bereaved and non-bereaved parents, and examined whether volunteering protects bereaved parents. We analyzed a pooled sample of parents aged 50+ (N = 12,023) from the Health and Retirement Study, including parents who lost a child prior to 50 and those who never lost a child. Bereaved parents reported more depressive symptoms and lower life satisfaction than non-bereaved parents, which was more evident among parents with fewer children alive. Among bereaved parents, volunteering, particularly volunteering 100+ hours/year, was associated with better well-being at baseline; yet, volunteering 1–99 hours/year led to a larger increase in life satisfaction over time. This study adds to our understanding of lasting effects of parental bereavement and suggests volunteering as a potential intervention aimed at helping bereaved older parents.

